# Supplementary material for: Novel Model to Predict HCC Recurrence after Liver Transplantation Obtained Using Deep Learning: A Multicenter Study
Source: Cancers (Basel). 2020 Sep 29;12(10):2791. doi: 10.3390/cancers12102791 (PMC7650768; doi:10.3390/cancers12102791)
Supplement: Supplementary file 1 [file cancers-12-02791-s001.zip › cancers-940658-supplementary.docx]

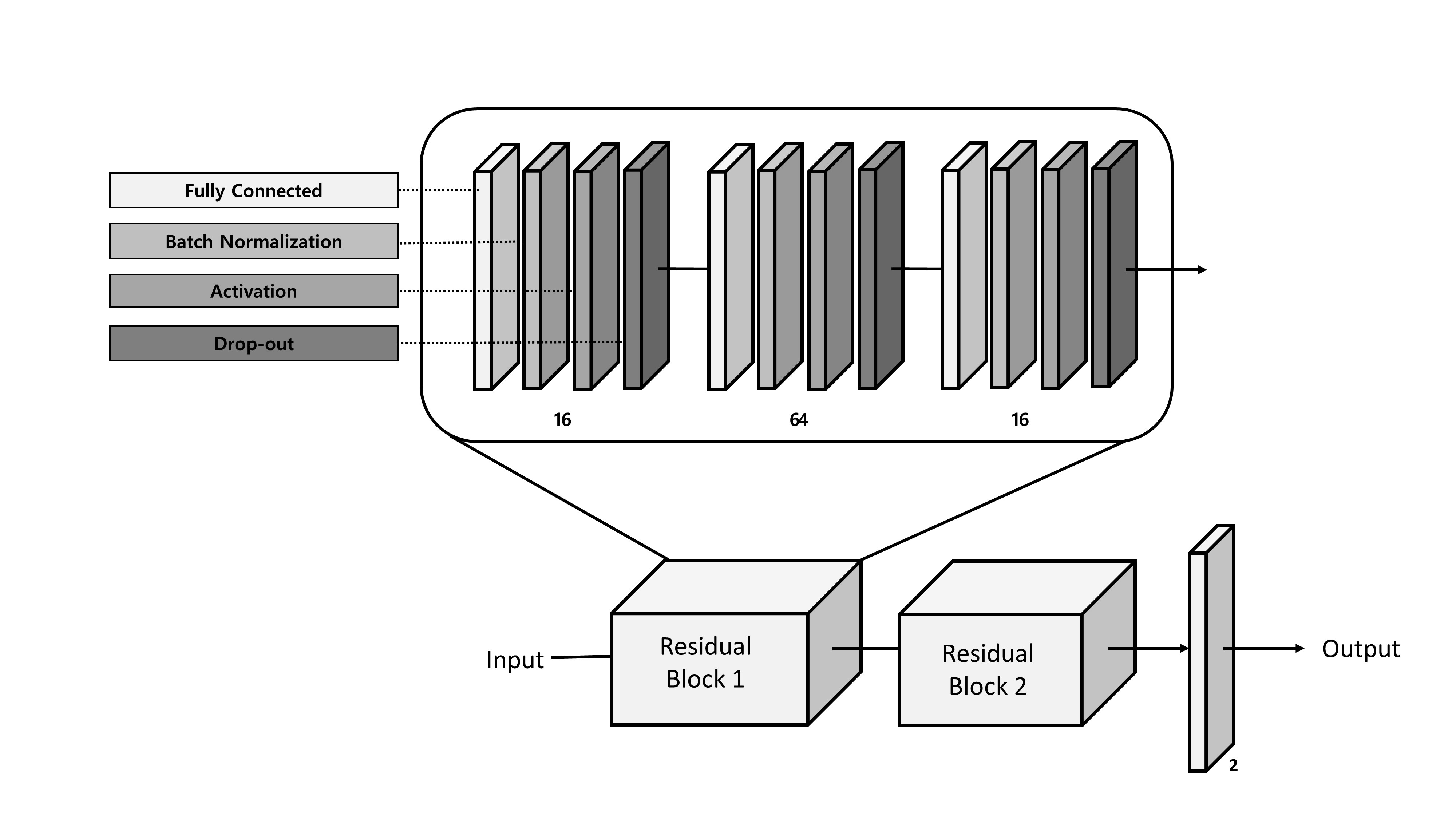


**Figure S1.** Network architecture.

**
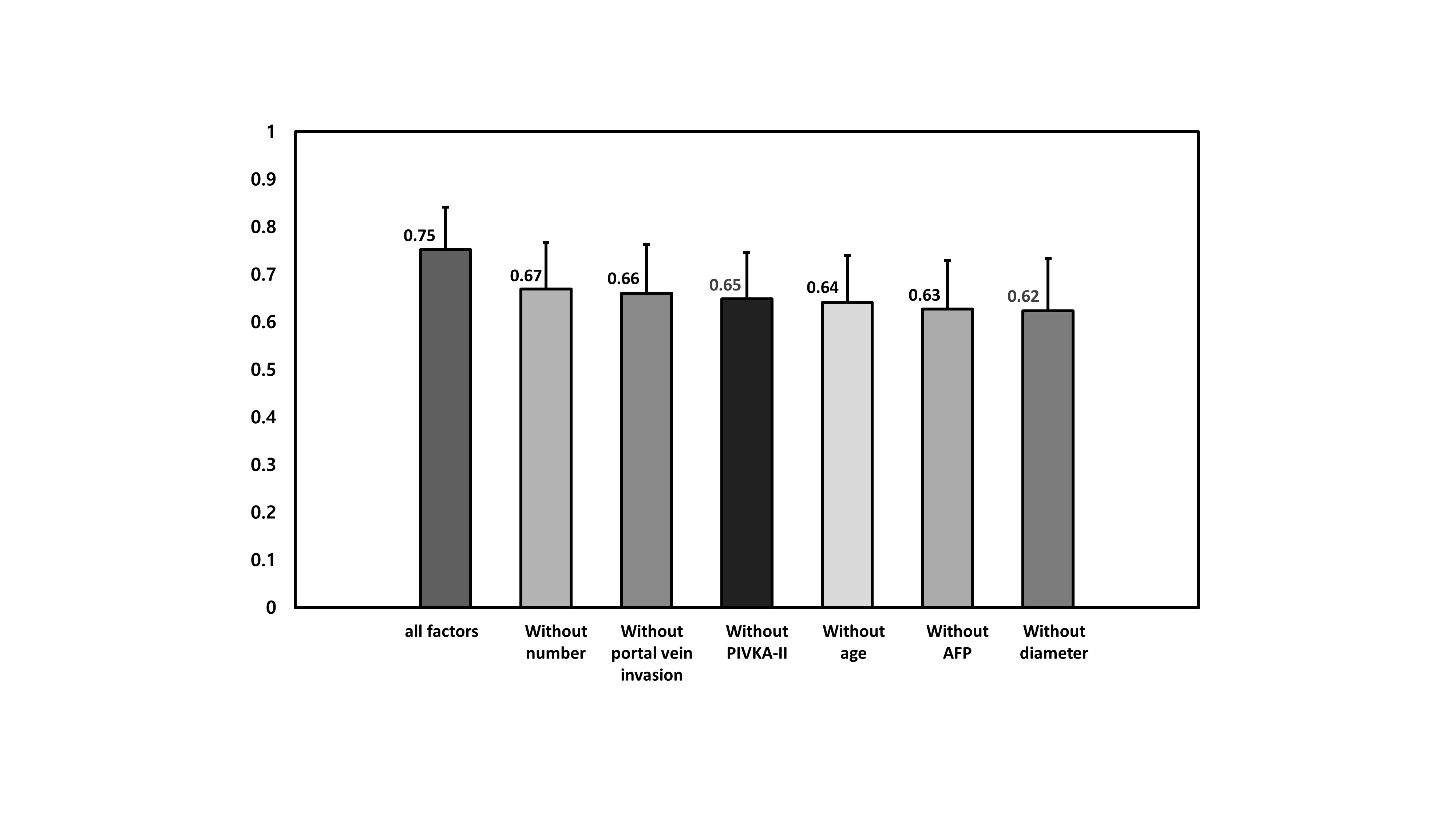
Figure S2.** C-index comparison among models without each factor in turns.
